# Progastrin Represses the Alternative Activation of Human Macrophages and Modulates Their Influence on Colon Cancer Epithelial Cells

**DOI:** 10.1371/journal.pone.0098458

**Published:** 2014-06-05

**Authors:** Carlos Hernández, María Dolores Barrachina, Jesús Cosín-Roger, Dolores Ortiz-Masiá, Ángeles Álvarez, Liria Terrádez, María Jesús Nicolau, Rafael Alós, Juan Vicente Esplugues, Sara Calatayud

**Affiliations:** 1 Departamento de Farmacología and CIBER, Facultad de Medicina, Universidad de Valencia, Valencia, Spain; 2 FISABIO, Hospital Dr. Peset, Valencia, Spain; 3 Unidad Mixta de Investigación en Biomedicina y Farmacología FISABIO - Hospital Dr.Peset - UVEG, Valencia, Spain; 4 Fundación General Universidad de Valencia, Valencia, Spain; 5 Hospital de Manises, Valencia, Spain; University of Pécs Medical School, Hungary

## Abstract

Macrophage infiltration is a negative prognostic factor for most cancers but gastrointestinal tumors seem to be an exception. The effect of macrophages on cancer progression depends on their phenotype, which may vary between M1 (pro-inflammatory, defensive) to M2 (tolerogenic, pro-tumoral). Gastrointestinal cancers often become an ectopic source of gastrins and macrophages present receptors for these peptides. The aim of the present study is to analyze whether gastrins can affect the pattern of macrophage infiltration in colorectal tumors. We have evaluated the relationship between gastrin expression and the pattern of macrophage infiltration in samples from colorectal cancer and the influence of these peptides on the phenotype of macrophages differentiated from human peripheral monocytes in vitro. The total number of macrophages (CD68+ cells) was similar in tumoral and normal surrounding tissue, but the number of M2 macrophages (CD206+ cells) was significantly higher in the tumor. However, the number of these tumor-associated M2 macrophages correlated negatively with the immunoreactivity for gastrin peptides in tumor epithelial cells. Macrophages differentiated from human peripheral monocytes in the presence of progastrin showed lower levels of M2-markers (CD206, IL10) with normal amounts of M1-markers (CD86, IL12). Progastrin induced similar effects in mature macrophages treated with IL4 to obtain a M2-phenotype or with LPS plus IFNγ to generate M1-macrophages. Macrophages differentiated in the presence of progastrin presented a reduced expression of Wnt ligands and decreased the number and increased cell death of co-cultured colorectal cancer epithelial cells. Our results suggest that progastrin inhibits the acquisition of a M2-phenotype in human macrophages. This effect exerted on tumor associated macrophages may modulate cancer progression and should be taken into account when analyzing the therapeutic value of gastrin immunoneutralization.

## Introduction

Macrophages are a significant component of tumors and display a variety of functions depending on the local environment. They can be pro-inflammatory and help to generate adaptive immune responses (classically activated macrophages, M1) or tolerogenic/anti-inflammatory (alternatively activated macrophages, M2) [1], [2]. Tumor associated-macrophages (TAMs) often resemble M2-macrophages and, therefore, instead of fighting against cancerous cells, these leukocytes promote tumor growth by dampening the immune response and through the secretion of growth and angiogenic factors as well as the enzymes necessary for cell invasion. As a consequence, the presence of TAMs has been correlated with a decreased survival in patients with e.g. melanoma, breast, kidney or bladder cancer. The situation seems to be different in some cancerous processes affecting the gastrointestinal tract. In patients with colorectal or gastric cancers a higher macrophage infiltration correlates with a better prognosis [3]. The reasons for this differential role of macrophages in these particular diseases is far from clear, but from the current knowledge one can infer that the colorectal/gastric tumor microenvironment marks a different equilibrium in the function of infiltrated M1 and M2 macrophages, with M2 macrophages exerting a defective opposition to the accompanying M1-phagocytes [4], [5]. However, the mechanisms responsible for this effect are unknown.

Most adenomatous polyps, colorectal and gastric tumors express ectopically the gastrin gene. However, these cancer cells are not of an endocrine nature and mainly synthesize the hormone precursor progastrin [6]–[8], which has proliferative action on cancer cells [9]. Although progastrin may bind with low affinity to gastrin CCK-2 receptors and this interaction may contribute to some extent to its biological activity [10], progastrin's growth-promoting effect appears to be mainly mediated by a non-conventional receptor recently identified as annexin II [9]. We have observed that gastrin exerts a pro-inflammatory activity through CCK-2 receptors [11]–[13], which are expressed in macrophages and endothelial cells, while annexin II is highly expressed on the surface of macrophages, where it serves as a pathogen recognition element and mediates macrophage activation [14].

Our hypothesis was that gastrin peptides locally produced in colonic tumors can influence the function of infiltrated macrophages and, in this way, modulate the immune response to disease. We observed that the expression of gastric peptides in colorectal tumor cells correlates with a reduced infiltration of M2-macrophages and showed that progastrin modulates the maturation process of human macrophages in vitro, and represses the acquisition of a M2-phenotype. Progastrin also reduced the secretion of Wnt ligands by M2-macrophages and increased their ability to induce apoptosis of colon cancer epithelial cells.

## Methods

### Patients

Twenty-one curatively resected colorectal carcinoma patients (Table 1) were selected randomly from patients operated at the Hospital de Manises. None of them had any preoperative radio/chemotherapy. Immediately after resection, a piece containing tumor and surrounding normal tissue was fixed in buffered formalin and embedded in paraffin. Experienced pathologists documented the histopathological characteristics of the tumors, including tumor stage, differentiation grade, size, lymph/angioinvasion, perineural invasion and lymph node involvement. Tumor stage was defined according to the TNM staging system. The study was approved by the Institutional Review Board of The Hospital of Manises (Valencia). Written informed consent was obtained from all patients.

**Table 1:** Characteristics of patients and tumors.

		N (%)
Sex	Female	7 (33)
	Male	14 (67)
Age	50–60 years	2 (10)
	60–70 years	9 (43)
	70–80 years	7 (33)
	>80 years	3 (14)
Site of primary tumor	Colon	15 (71)
	Rectum	6 (29)
Tumor differentiation	Grade 1	3 (14)
	Grade 2	17 (81)
	Grade 3	1 (5)
TNM stage	T2N0M0	3 (14)
	T3N0M0	10 (48)
	T3N1M0	1 (5)
	T3N1M1	1 (5)
	T3N2M0	2 (10)
	T3N2M1	1 (5)
	T4N0M0	1 (5)
	T4N1M0	1 (5)
	T4N2M0	1 (5)

### Inmunohistochemistry

Serial sections (4 µm) of samples containing tumor and normal mucosa from each patient were stained for gastrin, Wnt1, CD68 as a macrophage marker, CD86 as a M1-macrophage marker or CD206 as a M2-macrophage marker. Samples were subjected to different methods of antigen retrieval depending on the epitope (Table 2). Endogenous peroxidase activity was suppressed by immersion in 0.3% hydrogen peroxide. Once blocked with 5% horse serum, sections were incubated overnight (4°C) with the corresponding primary antibody (Table 2). A horse anti-mouse/rabbit biotinylated antibody (Vector Laboratories, CA, USA, 1∶200) was used as a secondary antibody. The VECTASTAIN elite ABC system Kit (Vector Laboratories, CA, USA), followed by the DAB Enhanced Liquid substrate System for Immunohistochemistry (Sigma-Aldrich, Missouri, USA) were used for development. All tissues were counterstained with hematoxylin and the specificity of the immunostaining was confirmed if analogous tissue sections showed an absence of staining after using non-immune immunoglobulin of the same isotype and at the same concentration as the primary antibody or after omitting the secondary antibody.

**Table 2:** Antibodies used in immunohistochemistry (IHC) and static cytometry studies.

Antigen	Technique	Primary antibody	Antigen retrieval treatment*
CD68	IHC	Monoclonal Mouse Anti-Human CD68 Clone PG-M1 (Dako)	α-Chymotrypsin (Sigma), 20 min, 37°C
CD86	IHC	B7-2/CD86 (Epitomics)	Target Retrieval Solution pH 6 (Dako), 20 min, 95°C
	Static cytometry	FITC Mouse Anti-Human CD86 (BD Pharmingen)	-
CD206	IHC	Anti-MRCI (Sigma)	Target Retrieval Solution pH 9 (Dako), 20 min, 95°C
	Static cytometry	PE Mouse Anti-Human CD206 (BD Pharmingen)	-
Gastrin	IHC	FLEX Polyclonal Rabbit Anti-Human Gastrin (Dako)	Target Retrieval Solution pH 9 (Dako), 20 min, 95°C
Wnt1	IHC	Polyclonal Rabbit Anti-Wnt1 (Sigma)	Target Retrieval Solution pH 6 (Dako), 20 min, 95°C

*Procedure to unmask the antigen.

A representative area (objective 40X, 6 fields, 0.22 mm^2^) from tumoral tissue or adjacent normal glandular tissue was selected for quantitative analysis of macrophage infiltration. Gastrin staining in each sample was evaluated and a score for intensity from 1 to 4 was assigned. Only cancer epithelial cells reacted to gastrin antibody and the intensity of the staining was very homogeneous through the entire epithelial tumor compartment. All samples were processed in parallel and an observer, unaware of the patient number and different from the person who processed and analyzed the macrophage immunostainings, assigned an score to each patient based on the intensity of staining observed in three pictures representative of different parts of the tumor.

### Cell culture

Human peripheral blood mononuclear cells were isolated from healthy donors by Ficoll density gradient centrifugation. Monocytes were seeded in tissue culture plates and matured to macrophages by culturing in X-Vivo 15 medium (BioWhittaker) supplemented with 1% human serum and 20 ng/nl of recombinant human M-CSF (Peprotech) at 37°C in 5% CO_2_ for up to 6 days. In order to obtain an M1 polarization, cells were incubated with 1 µg/ml LPS (from Escherichia coli 0111:B4, Sigma-Aldrich) plus 20 ng/ml human recombinant IFNγ (Peprotech) for the last 24 hours. M2 polarization was obtained by treating cells with 20 ng/ml of human recombinant IL4 (Peprotech) for the last 48 hours of the culturing period. The maturation process as well as the activation treatments were carried out in the presence or absence of different concentrations of progastrin (10^−11^–10^−7^M, Abgent).

Caco-2 cells (American Type Culture Collection, VA, USA) were cultured in MEM medium (Sigma-Aldrich) supplemented with 20% inactivated bovine foetal serum, 100 U/ml penicillin, 100 µg/ml streptomycin, 2 mM L-glutamine, 100 mM sodium pyruvate and 1% of non-essential amino acids.

Caco-2 cells were co-cultured with monocyte-derived macrophages using Transwell inserts (Corning Incorporated, MA, USA) with a 0.4 µm porous membrane [12]. Monocytes were seeded on these inserts and differentiated to macrophages in the presence or absence of different concentrations of progastrin. On day 6 after seeding, the inserts were placed on top of Caco-2 cells (t = 0) and were maintained in co-culture for 24 hours.

The presence of the surface molecules CD86 (M1) and CD206 (M2) in cultured macrophages was analyzed by fluorescence microscopy (microscope IX81, Olympus, Hamburg, Germany). Macrophages were incubated with hoescht to identify the nuclei and with fluorescent labeled antibodies against these targets (Table 2). Fluorescence was analyzed with the static cytometry software ScanR (>5000 cells/well).The concentration of the cytokines IL12 (Th1) and IL10 (Th2, antiinflammatory) in cell supernatants was determined by ELISA (Diaclone).

The number of Caco-2 cells after co-culture was counted in a Newbauer chamber. Apoptosis in these cells was studied by flow cytometry as bivariate Annexin V/PI analysis (Apoptosis Detection Kit, Abcam).

Total RNA from macrophages or Caco-2 cells was isolated by using the extraction kit (Illustra RNAspin Mini, GE HealthCare Life Science) and cDNA was obtained with the Prime Script RT reagent Kit (Takara Biotechnology). Real-time PCR was performed with the Prime Script Reagent Kit Perfect Real Time (Takara Biotechnology) in a thermo cycler LightCycler (Roche Diagnostics, Mannheim, Germany). Specific oligonucleotides were designed according to sequences shown in Table 3. Relative gene expression was expressed as previously described [15].

**Table 3:** Primer sequences of specific PCR products for each gene analyzed.

Human Gene	Sense	Antisense	Length (bp)
*Wnt1*	5′-CGCCCACCCGAGTACCTCCA-3′	5′-TTCATGCCGCCCCAGGCAAG-3′	110
*Wnt3a*	5′-TACTCCTCTGCAGCCTGAAGCA-3′	5′-ATGGCGTGGACAAAGGCCGAC-3′	322
*Wnt5a*	5′-CTGCCCCAACTCGGGAGTCCAGG-3′	5′-AGGAATCCGAGCGGAGCGACC-3′	147
*Lgr5*	5′-GGCTCGGTGTGCTCCTGTCCT-3′	5′-TGCCTCAGGGAATGCAGGCC-3′	484
*β-actin*	5′-GGACTTCGAGCAAGAGATGG-3′	5′-AGCACTGTGTTGGCGTACAG-3′	67

### Statistical analysis

Data are expressed as mean ±s.e.m. and were compared by analysis of variance (one way-ANOVA) with a Newman-Keuls post-hoc correction for multiple comparisons or a t-test when appropriate. A P value <0.05 was considered to be statistically significant. The clinical correlations in human samples were analyzed using the Pearson's correlation coefficient.

## Results

### M2 macrophage number is increased in the tumoral area

**Figure 1:**
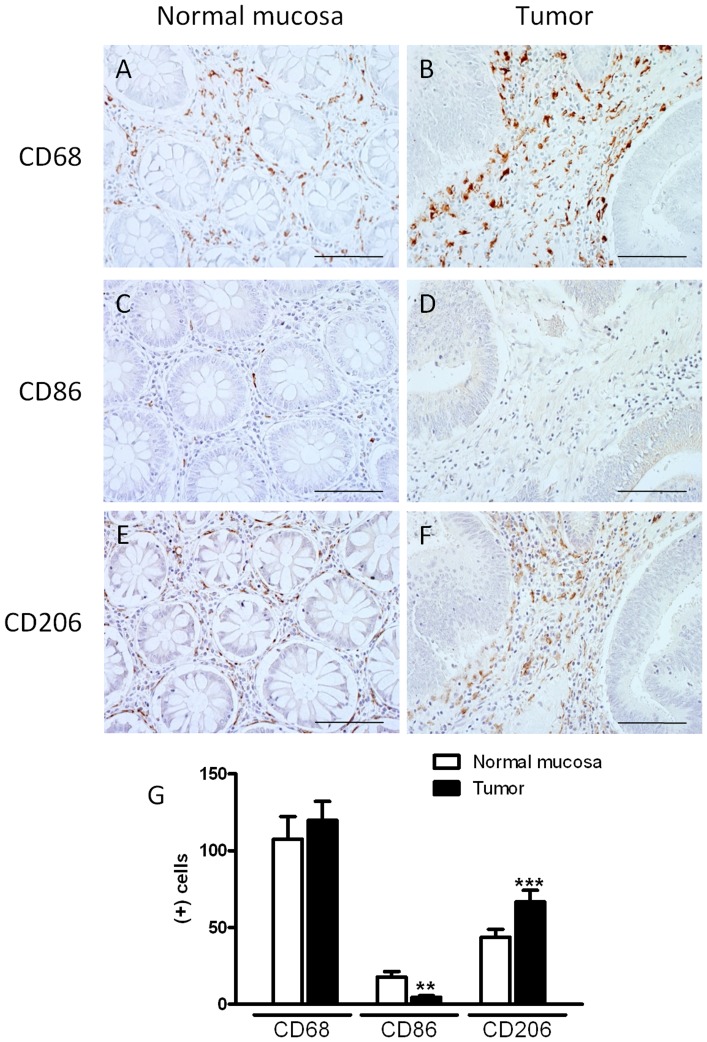
Pattern of macrophage infiltration in colorectal cancer and healthy surrounding tissues. The immunoreactivity to CD68 (MF marker, A–B), CD86 (M1-MF marker, C–D) and CD206 (M2-MF marker, E–F) was analyzed in colorectal cancer (B, D, F) and healthy surrounding mucosa (A, C, E). (G) Quantitative analysis of positive cells for these molecules in a representative area of 0.22 mm^2^ (Scale bar  = 0.2 mm). Bars represent mean ±SEM (n = 21). **P<0.01 and ***P<0.001 vs corresponding value in normal mucosa.

We first analyzed the amount and phenotype of macrophages in the non-tumor and tumor areas of colorectal cancer patients. Immunohistochemistry for CD68 (macrophage marker), CD86 (M1 macrophage marker) and CD206 (M2 macrophage marker) was performed in biopsy samples from colorectal carcinoma resections. Of note, the number of macrophages was similar in tumor and normal surrounding tissue, but the number of M1 and M2 macrophages was significantly different between the two areas (Figure 1). Tumor stroma contained a lower number of CD86(+) cells than lamina propria of the normal tissue. In contrast, a higher number of CD206(+) cells was encountered in tumoral tissue compared with normal surrounding tissue, suggesting that tumor development favors the differentiation of monocytes to M2 macrophages.

### Gastrin expression is increased in the tumor but negatively correlates with the number of M2 macrophages

**Figure 2:**
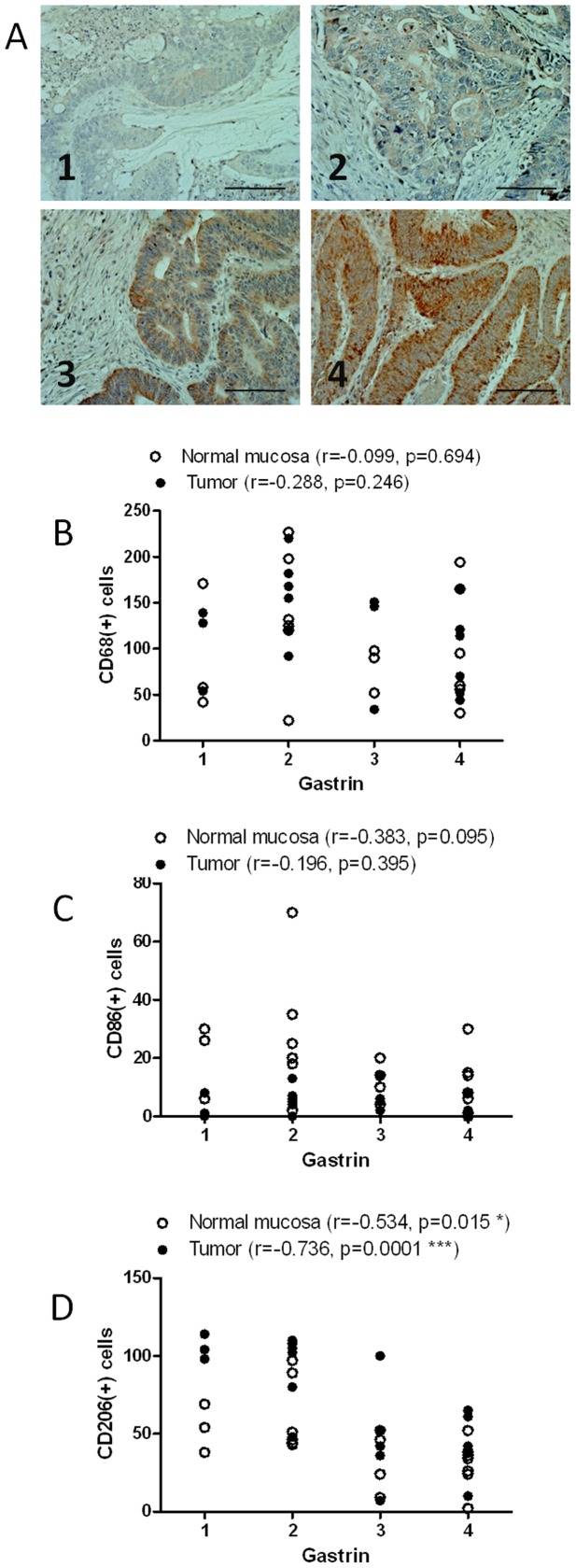
Relationship between gastrin expression and the pattern of macrophage infiltration in colorectal cancer samples. The number of total macrophages (CD68 + cells, B), M1-macrophages (CD86+ cells, C), and M2-macrophages (CD206 cells, D), in the tumor and normal surrounding tissue were analyzed in relation to gastrin expression in tumoral tissue (A, score 1 to 4, scale bar  = 0.2 mm). A negative and significant correlation is observed between the intensity of gastrin staining and the number of M2-macrophages in normal mucosa and tumoral tissue (D).

Searching for mediators that could modulate the phenotype of macrophages into the tumor we studied the role of gastrin peptides on the expression of different macrophage markers in the biopsy samples. Since the gastrin antibody used in this study recognizes both gastrin and gastrin precursors, like progastrin, we cannot discriminate between different gastrin peptides, but we observed extensive immunoreactivity in tumor epithelial cells of biopsies analyzed. Staining in each sample was evaluated and a score for intensity from 1 to 4 was assigned (Figure 2A). No correlation was observed between gastrin levels in tumors and the number of total (CD68+ cells) or M1 (CD86+ cells) macrophages either in the tumor or in normal tissue (Figures 2B and 2C). Surprisingly, the number of M2 macrophages in tumoral and normal tissue correlated negatively with gastrin expression. This correlation was more significant in the tumor, where the peptide is synthesized. Taken together these data suggest that gastrin peptides synthesized by tumor cells reduces the number of M2 macrophages, without affecting the total macrophage number.

### Progastrin decreases the differentiation of macrophages towards a M2 phenotype

**Figure 3:**
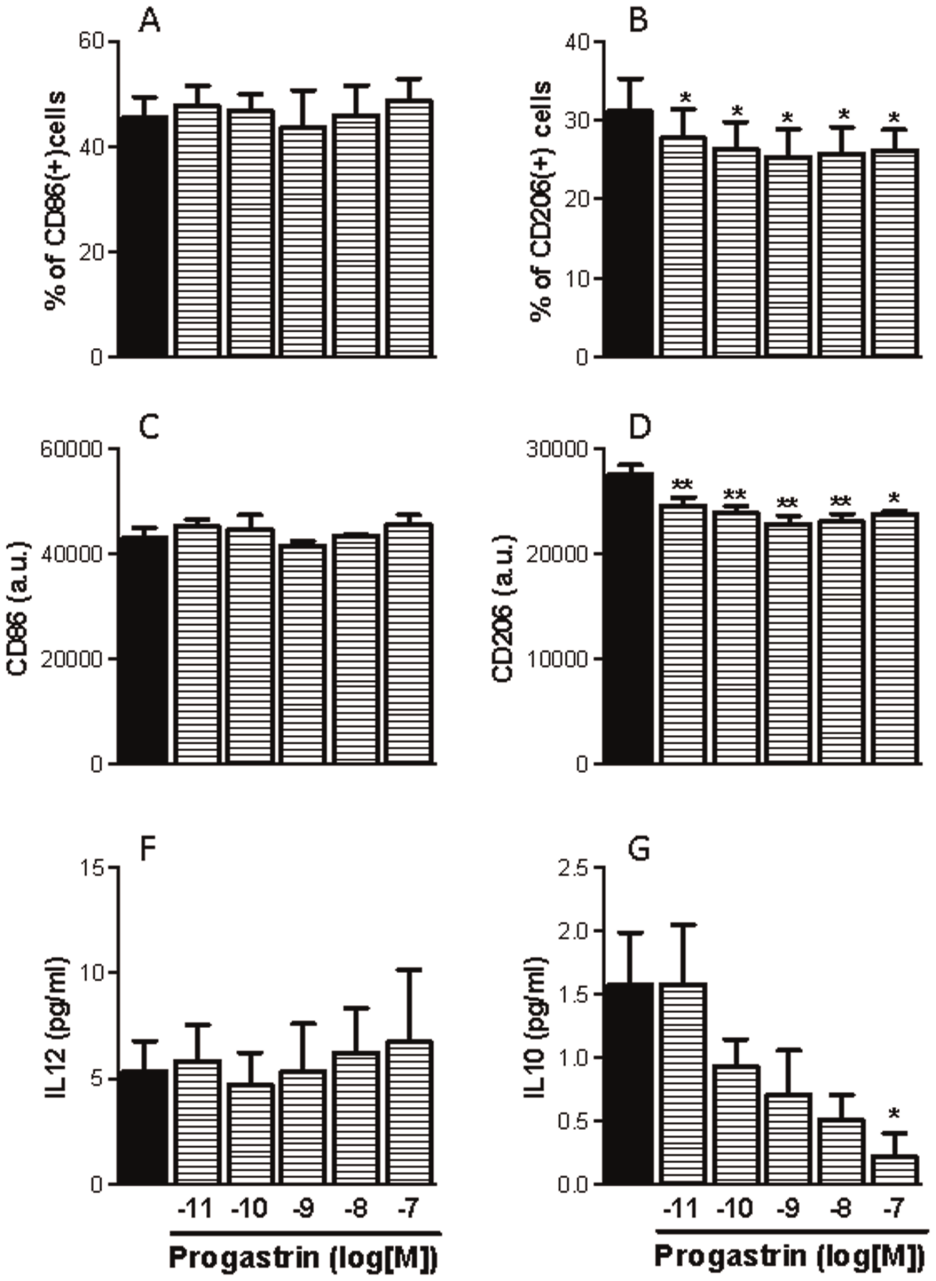
Influence of progastrin on the phenotype of human monocyte-derived macrophages. Human peripheral monocytes were derived to macrophages in the presence or absence of progastrin and the phenotype of the resultant macrophages evaluated by analyzing the following parameters: expression of CD86 (A, C, n = 3); expression of CD206 (B, D, n = 3); secretion of IL12 (F, n = 4); and secretion of IL10 (G, n = 4). Bars represent mean ±SEM. *P<0.05 and **P<0.01 vs corresponding value in vehicle-treated cells.

We hypothesized that progastrin could be responsible for the effects observed on macrophage phenotype, because colon cancer cells lack the enzymes necessary for complete gastrin maturation [7]. To investigate whether progastrin can modulate the differentiation of macrophages to a M1- or M2-phenotype we treated human peripheral monocytes obtained from healthy volunteers with several concentrations of progastrin and left them to differentiate. Static cytometry experiments carried out with specific fluorescent antibodies showed that progastrin reduced the expression of the M2-marker CD206 in monocyte-derived macrophages even at the lowest concentration analyzed. In contrast, no changes in the expression of the M1-marker CD86 were detected. In addition, we measured the concentration of IL-12 and IL-10 in the supernatant of monocyte-derived macrophages after 6 days of differentiation in the presence of increasing doses of progastrin. The gastrin precursor elicited a significant reduction of IL-10 secretion but did not alter the IL-12 levels (Figure 3). Our data indicate that progastrin inhibits the expression of M2-markers during macrophage differentiation without affecting the expression of M1-markers.

**Figure 4:**
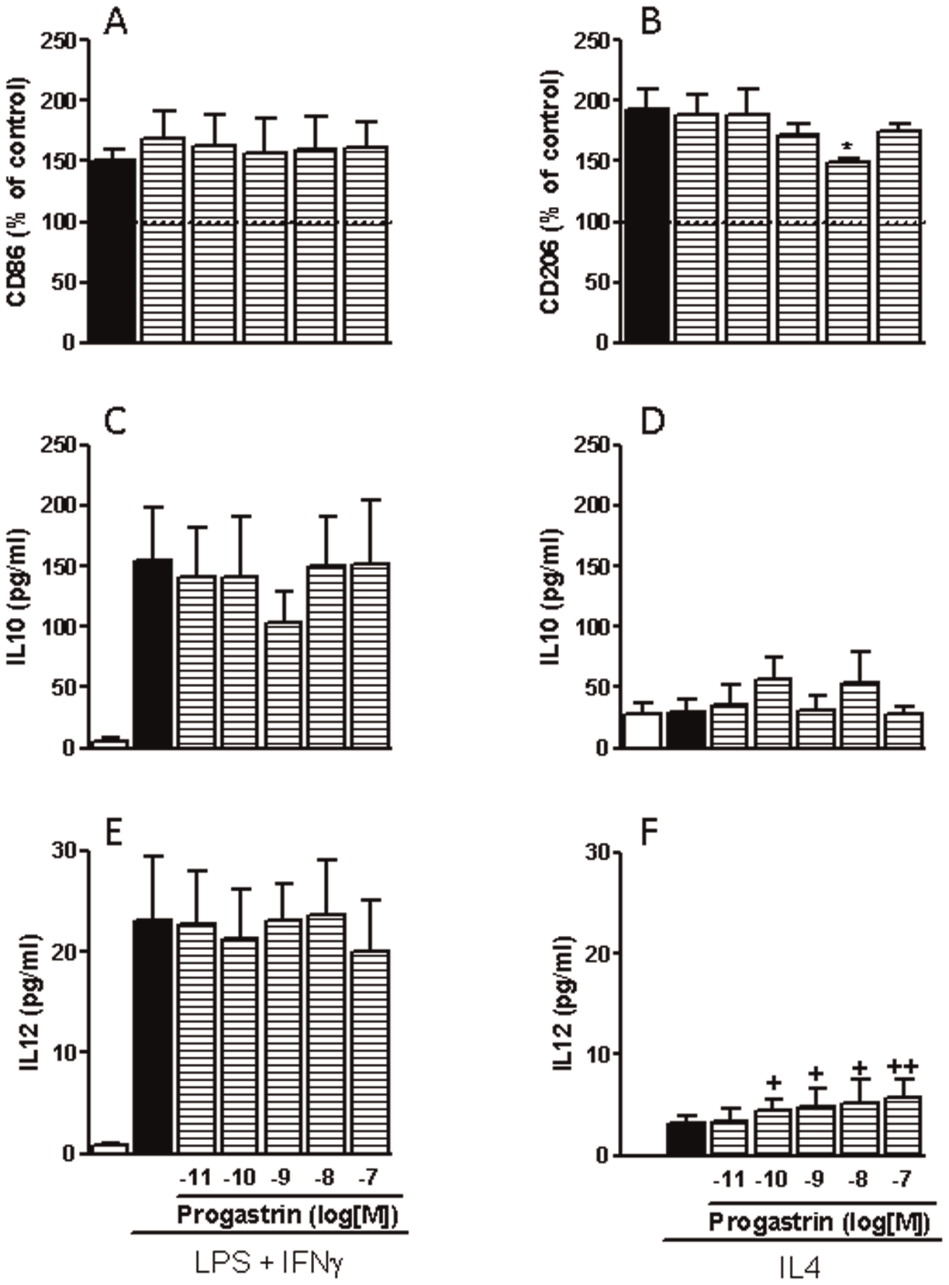
Influence of progastrin on human M1 and M2 macrophages. Human monocyte-derived macrophages were stimulated with LPS plus IFNγ to obtain a M1-phenotype or with IL4 to obtain M2-macrophages in the presence or absence of progastrin, and the resultant phenotype evaluated by analyzing the following parameters: expression of CD86 (A, n = 3); expression of CD206 (B, n = 5); secretion of IL10 (C, D, n = 5); and secretion of IL12 (E, F, n = 5). Bars represent mean ±SEM. *P<0.05 vs corresponding value in vehicle-treated cells; ^+^ P<0.05 and ^++^P<0.01 vs corresponding value in control cells.

In order to study the effect of progastrin on IL4-mediated differentiation of macrophages towards a M2-phenotype we incubated fully differentiated monocyte-derived macrophages with IL-4 and different doses of progastrin for two days. Basal macrophages treated with IL-4 showed a significant increase in CD206 expression, a non-significant increase in IL-12 secretion and similar IL-10 production than controls. Progastrin significantly reduced the induction of CD206 by IL-4 and increased IL-12 secretion to levels significantly higher than those observed in control cells, while secretion of IL-10 remained unchanged. However, differentiation of macrophages towards a M1-phenotype was not affected by progastrin. Treatment with LPS plus IFNγ increased the expression of CD86, and the secretion of both IL12 and IL10 in macrophages, but progastrin did not modify any of these parameters (Figure 4).

### Progastrin down-regulates Wnt ligands in macrophages and increases cell death in co-cultured Caco2 cells

**Figure 5:**
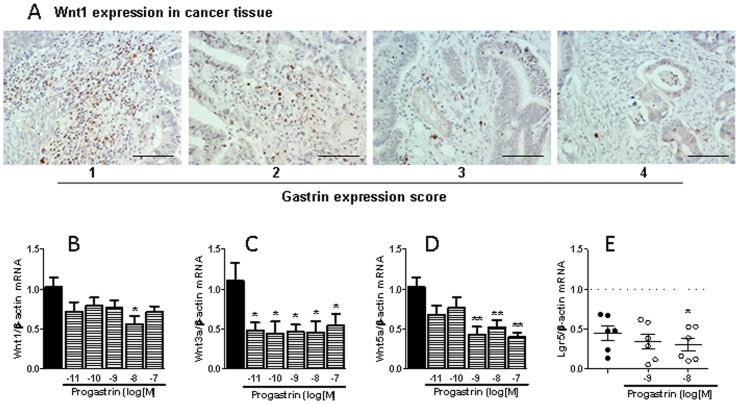
Influence of progastrin on macrophage-derived Wnt ligands. Immunoreactivity to Wnt1 in the stroma of colorectal cancer in relation to the expression of gastrin in the same tissue (scale bar  = 0.2 mm) (A); and effects of the presence of progastrin during differentiation of human peripheral monocytes to macrophages on mRNA expression of three different Wnt ligands in these macrophages (B–D, n = 5) and mRNA expression of Lgr5 in co-cultured Caco-2 cells (E, n = 7). Bars represent mean ±SEM. *P<0.05 and **P<0.01 vs corresponding value in vehicle-treated cells.

Next we analyzed whether the modulation of macrophage phenotype by progastrin could affect the surrounding tumor cells. We have recently reported that M2-macrophages synthesize and secrete Wnt ligands which can modulate co-cultured epithelial cell behavior [16] and in the present study we observed immunoreactivity for Wnt1 in cells of the tumor stroma. Qualitative assessment of the expression of this Wnt ligand indicates that the amount of positive cells decreases as gastrin production in cancer cells increases (Figure 5A). A causal relationship between both factors is pointed out by our in vitro studies showing that macrophages maturated in the presence of progastrin present a significantly reduced mRNA expression of three different Wnt ligands (Wnt1, Wnt3a, and Wnt5, Figures 5B–5D). Functional relevance of the down-regulation of the three Wnt ligands in macrophages is demonstrated by the fact that Caco2 cells co-cultured with progastrin-treated macrophages expressed less Lgr5 mRNA than Caco2 cells co-cultured with control macrophages (Figure 5E). These data indicate that the effects of progastrin on macrophage-derived Wnt ligands modulate the Wnt signaling pathway in surrounding tumor cells.

**Figure 6:**
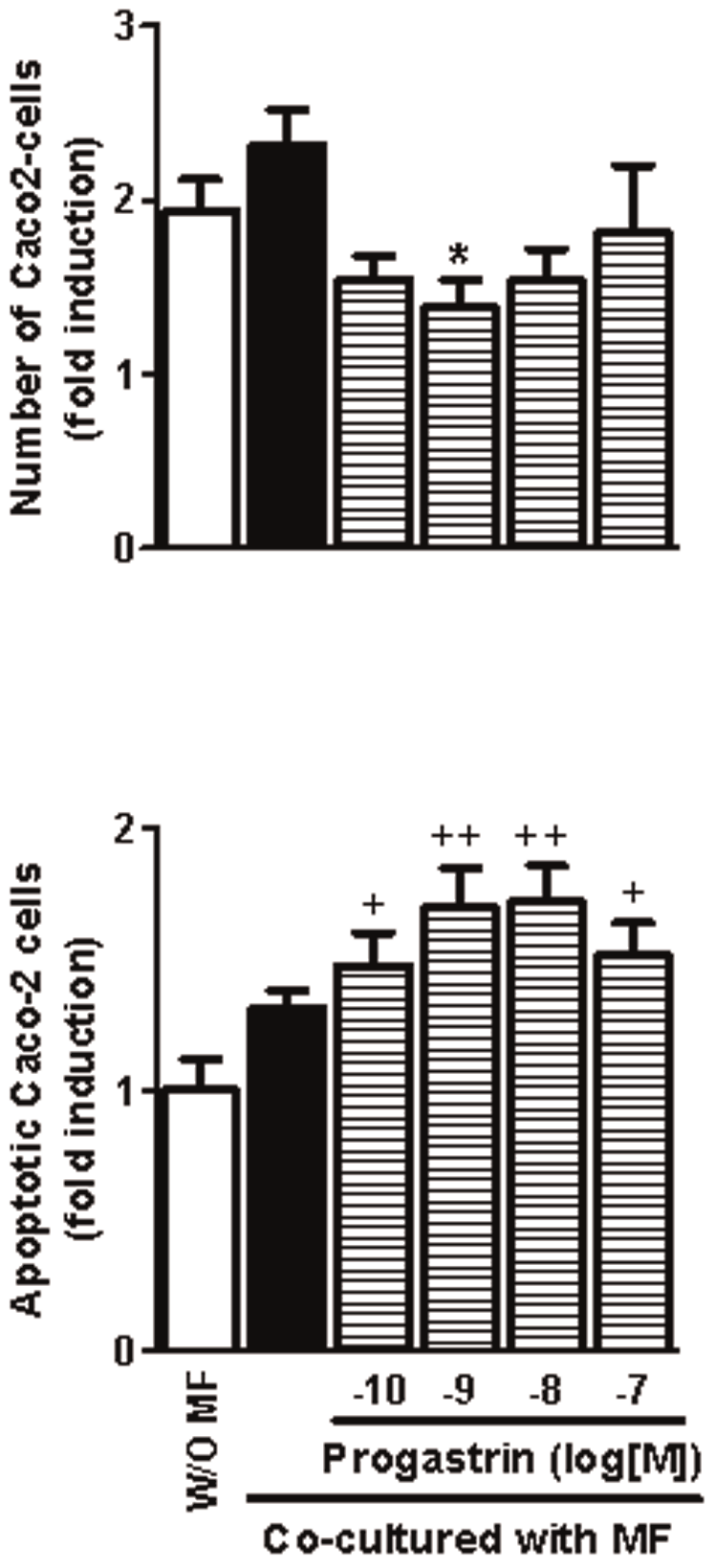
Influence of progastrin-treated macrophages on colorectal cancer cells. The effects of human monocyte-derived macrophages obtained in the presence or absence of progastrin on the number (A) and the rate of apoptosis (B) of Caco-2 cells were analyzed in a co-culture system (24 h, n = 4). Bars represent mean ±SEM. *P<0.05 vs corresponding value in vehicle-treated macrophages, ^+^P<0.05 and ^++^P<0.01 vs cells incubated with an empty insert (w/o MF).

Furthermore, co-culture of Caco-2 with progastrin-treated macrophages resulted in a significant reduction in the total number of epithelial cells together with an increased rate of apoptosis (Figure 6).

## Discussion

This study shows that macrophages infiltrating colorectal tumors have a different phenotypic profile than those that are present in the normal tissue surrounding the cancerous lesion, with a higher proportion of the anti-inflammatory M2-macrophages and significantly lower numbers of classically activated M1-macrophages. Interestingly, our results indicate that gastrin expression in tumoral tissue exerts a negative influence on the number of tumoral M2-macrophages.

The expression of gastrin in tumoral epithelial cells correlates negatively with the number of tumoral M2-macrophages and our in vitro results suggest a causal relationship between both factors. Human macrophages obtained by culturing peripheral monocytes in the presence of progastrin showed a reduced expression of CD206. Moreover, this peptide significantly prevented the expression of CD206 when mature macrophages were stimulated with the Th2 cytokine IL4 to induce an M2-phenotype. In contrast, progastrin did not affect the expression of the co-stimulatory molecule CD86 either in resting conditions or in macrophages stimulated with LPS plus IFNγ to develop an M1-phenotype. Progastrin also affects the pattern of cytokine secretion. Macrophages maturated in the presence of this peptide released lower amounts of the anti-inflammatory cytokine IL10 while maintaining normal release of the Th1 inducer IL12. In IL4-stimulated macrophages, the effects on cytokine secretion were different but in the same direction. In this case, IL10 release was unaffected while the peptide facilitated IL12 secretion. Thus, it is expectable that those macrophages that are under the influence of progastrin in the cancerous tissue present a blunted anti-inflammatory, immunosuppressive profile.

This effect is in line with the previously described proinflammatory action of gastrin [11]–[13]. We observed that gastrin contributes to the inflammation induced by Helicobacter pylori in rats probably through CCK-2 receptor activation in macrophages while the stimulation of this receptor activates human endothelial cells to promote monocyte adhesion. CCK-2 receptors can bind all gastrin peptides, although their affinity for mature gastrin is significantly higher than that demonstrated for progastrin. The actions of the latter may be alternatively transmitted by the recently characterized non-conventional receptor annexin-II [9]. Both kinds of receptors are present in macrophages and both pathways promote macrophage activation [14]. Thus, the present results reinforce the notion that gastrin peptides tend to contribute to inflammation although different mechanism may be involved in each case.

The influence of macrophages on cancer progression is due to their effect on the immune response against the tumor but also to a direct local effect on the surrounding epithelial cancer cells. Macrophages can be a source of Wnt ligands [17], the oncogenic pathway activated in the majority of colorectal cancers and a significant contributor to cancer cell stemness [18], [19]. Secretion of these factors is especially relevant in a subpopulation of TAMs that seem to play a particular role in tumor invasiveness by promoting angiogenesis and tumoral cell migration through Wnt-signaling [20], [21]. Wnt1 was detected in cells of the tumor stroma and its presence tended to decrease, as the level of gastrin in the tumor increased. We have recently observed that secretion of Wnt ligands is specifically increased in M2 macrophages, which promote Wnt/β-catenin signaling pathway and the consequent proliferative activity in co-cultured colon cancer cells [16]. Our present results show that macrophages maturated in the presence of progastrin express lower amounts of three different Wnt ligands and provoke a significant reduction in the number of co-cultured Caco-2 cells. This effect occurs with a concomitant decrease in the expression in these colonic cells of Lgr5, a Wnt target gene [22] that potentiates Wnt/β-catenin signaling [18]. Moreover, macrophages maturated in the presence of progastrin tend to increase the apoptosis rate in Caco-2 cells, an effect that may also be related with reduced Wnt signaling [23]. Thus, the phenotypical changes induced by progastrin in macrophages modify their influence on colon cancer epithelial cells resulting in a diminished number of these cells probably by a combination of reduced proliferation and increased apoptotic cell death. Additionally, the reduced production of Wnt ligands induced by progastrin may also contribute to the described phenotypical changes induced by this peptide since an autocrine effect of Wnt5a in macrophages to reduce the expression of IL12 in response to bacterial products has been described [24].

From our results we can infer that gastrins, by inhibiting the acquisition of an M2-phenotype in local macrophages, may regulate cancer progression. It is clear that M2 macrophages stimulate the growth of colon-cancer epithelial cells in vitro and experimental studies demonstrate that M2-macrophages in the tumor promote colon cancer in mice [25]–[27]. In human colorectal cancers, the effect of M2-macrophages seems more complicated and affected by several factors like their spatial distribution [4], the relative amount of M1 macrophages [5] or the stage of the disease [4]. Although their presence have been seen as a negative prognostic factor by some authors [28], others suggest that M2 macrophages have a less hazardous effect in human colorectal cancer than in other settings and point to the idea that some local factor specifically present in this kind of tumors may be down-regulating their tumor promoting action [4], [5], [29]. Keeping in mind that M1 and M2 macrophages are not clonally different sets of cells but the extremes of a wide spectrum of intermediate phenotypes and that classification of a cell as an M2 macrophage may be biased by the molecular marker selected in each case, we launch the idea that those macrophages identified as M2 in these studies may be less deleterious because of progastrin.

A direct proliferative function of progastrin on epithelial cells has been clearly demonstrated in isolated cells and mice, and experimental studies with transgenic animals suggest that overexpression of gastrins in the presence of DNA-damaging agents enhances carcinogenesis. This evidence has prompted several pharmacological strategies to neutralize the activity of gastrin peptides, with successful results in murine models but, unfortunately, few and indefinite results in patients [30]. Our findings suggest that, at least in humans, progastrin can play a multifaceted role in the progression of colorectal tumors, as its proliferative activity on epithelial cells would be opposed by a potentially anti-tumoral action exerted on macrophages. The relevance of this effect on immune cells for the global activity of progastrin awaits evaluation by future research.

## Conclusions

Our study indicates that gastrin peptides synthesized by colon cancer cells have macrophages as their targets and probably affect the inflammatory infiltrate of the tumor, which in turn affects cancer progression. The inhibition of M2-polarization induced by progastrin would counteract their proliferative activity and, although it is difficult to estimate the magnitude of this effect, this observation should be taken into account when analyzing the therapeutic value of gastrin immunoneutralization [31].
